# Ajuba transactivates N‐cadherin expression in colorectal cancer cells through interaction with Twist

**DOI:** 10.1111/jcmm.16731

**Published:** 2021-06-26

**Authors:** Zhaoxia Wu, Xiuqun Zou, Ying Xu, Fengli Zhou, Rong Kuai, Ji Li, Daming Yang, Yimin Chu, Haixia Peng

**Affiliations:** ^1^ Digestive Endoscopy Center Key Laboratory for Translational Research and Innovative Therapeutics of Gastrointestinal Oncology Hongqiao International Institute of Medicine Tongren Hospital Shanghai Jiao Tong University School of Medicine Shanghai China; ^2^ Department of Biochemistry & Molecular Cellular Biology Shanghai Jiaotong University School of Medicine Shanghai China; ^3^ Digestive Endoscopy Center Shanghai Tongren Hospital Shanghai Jiaotong University School of Medicine Shanghai China

**Keywords:** acetylation, Ajuba, LIM domain, p300/CBP, Twist

## Abstract

Ajuba is a multiple LIM domain‐containing protein and functions as a transcriptional coregulator to modulate many gene expressions in various cellular processes. Here, we describe that the LIM domain of Ajuba interacts with Twist, and the Twist box is a pivotal motif for the interaction. Biologically, Ajuba enhances transcription of target gene *N‐cadherin* as an obligate coactivator of Twist. The enhancement is achieved by binding to the E‐box element within *N‐cadherin* promoter as revealed by luciferase reporter and chromatin immunoprecipitation assays. Mechanistic investigation demonstrates that Ajuba recruits CBP and Twist to form a ternary complex at the Twist target promoter region and concomitantly enhances histone acetylation at these sites. These findings identify that Twist is a new interacting protein of Ajuba and Ajuba/Twist/CBP ternary complex may be a potential treatment strategy for Twist‐related tumour metastasis.

## 1 INTRODUCTION

The Ajuba adaptor or scaffold protein belongs to the Zyxin/Ajuba family of LIM proteins.^1^, ^2^ The family contains Ajuba, Limd1, Wtip, Zyxin, Lpp and Trip6. These proteins have the same structural characteristics, which are the preLIM domain at their amino‐terminus and two or three tandem LIM motifs at their carboxyl‐terminal region. The preLIM domain is diverse in sequence and abundant in glycine and proline residues and constitutes an SH3 consensus recognition site. The LIM motif is a cysteine‐rich, double zinc finger domain and primitively discovered in three critical transcriptional factors including Caenorhabditis elegans Lin‐11, rat Isl‐1 and Mec‐3, which regulate cell fate determination and differentiation.^1^, ^3^ LIM domain is highly conservative and mediates protein‐protein interaction as a diverse protein module.^4^

In consistence with its feature to shuttle between cytoplasm and nucleus, Ajuba contains a nuclear localization sequence (NLS) in the LIM domain and nuclear exporting sequence (NES) in the preLIM domain.^5^ Therefore, Ajuba can participate in assembling various protein complex and be implicated in a series of cellular processes such as cytoskeletal organization, cell‐cell adhesion, regulation of gene transcription, mitosis, cell differentiation, proliferation and migration in cytoplasm and nucleus.^6^ In nucleus, Ajuba can act as a transcriptional corepressor or coactivator to take part in transcriptional regulation. For example, Ajuba acts as a scaffold protein to recruit 14‐3‐3 and PRMT5 to Snail to repress E‐cadherin expression.^7^, ^8^ Comparably, Ajuba can interact with PPARγ using its non‐NR box element within the preLIM region to coactivate the transcriptional ability of PPARγ.^9^

In recent years, a series of investigation showed that Ajuba exerts a critical role in epithelial‐mesenchymal transition (EMT) and cancer metastasis.^10^, ^11^ Moreover, Ajuba can function as a Snail corepressor to modulate EMT and metastasis.^5^ EMT is known to be critical for embryonic development, tissue regeneration and tumour metastasis.^12^

Twist, a basic helix‐loop‐helix (bHLH) transcription factor, facilitates tumour invasion and metastasis by boosting EMT of cancer cells. During the EMT, Twist induces the transcription of genes encoding mesenchymal markers, such as fibronectin and N‐cadherin.^13^ In prostate carcinoma cells, Twist transactivates N‐cadherin expression by directly binding to an E‐box regulatory element within its promoter.^14^ However, the underlying mechanism of transcriptional activation by Twist is little known. In this paper, we report that Ajuba functions as the coactivator of Twist to activate the transcription of N‐cadherin.

## 2 MATERIALS AND METHODS

### 2.1 Cell lines and cell culture

HEK 293T, SW1116 and SW480 cells were obtained from ATCC and authenticated by DNA typing, and no signs of mycoplasma contamination were found. The cells were seeded in Dulbecco's modified Eagle's medium (Gibco, *Cat#*11005500T8) supplemented with 10% fetal bovine serum (Sigma, *Cat#*F2442) and incubated at 37°C with 5% CO_2_.

### 2.2 Plasmids, shRNA and lentiviruses

Plasmids of pMEX‐Myc‐Ajuba and their truncations have been described previously.^8^ The murine Twist cDNA was amplified from pBabe‐Flag‐Twist and digested with BamHI and XbaI, then inserted into the pcDNA3.1‐N‐Flag vector to create a Flag‐tagged Twist fusion protein in the N terminus. The Twist truncation mutants were subcloned into pcDNA3.1‐N‐Flag vector by the PCR method. The sequence of N‐cadherin gene promoter was obtained as described previously.^15^ The first intron (+746‐+3156 bp) from genomic DNA was amplified by PCR and subcloned into pGL3‐basic luciferase reporter plasmids. The primer set is as follows: 5'‐ AAATTTG‐AGCTCGGCTCTAGGGGCTGGATT‐3' (forward) and 5'‐GGTTGGAGATCTTGTTGTTCGGG‐CGTGTAA‐3' (reverse). The site‐directed mutagenesis reagents following the manufacturer's protocol (Stratagene, *Cat#* 200 518) were used for the point mutations of the Twist box of Twist and E‐box of N‐cadherin‐Luc reporter. For shRNA in SW1116 and SW480 cells, the following oligonucleotides were inserted into pLKO.1‐puro vector: 5'‐GCTCCTTATCTGTCTGA ‐GAAT‐3' for human *Ajuba* and 5'‐GCATTCTGATAGAAGTCTGAA‐3' for human *Twist*. All inserted fragments and cDNA of truncations and mutants were verified by DNA sequencing.

### 2.3 Cell transfections and lentiviral infection

Lentiviral supernatants for shRNA were produced by cotransfecting 293T cells with the construct pLKO.1‐shAjuba or pLKO.1‐shTwist and packing plasmids pMD2.G and psPAX2 by using X‐tremeGENE 9 DNA Transfection Reagent (Roche, *Cat#* 06365787001). Viral supernatant was collected at 24 ~ 48 hours post‐transfection, passed through a 0.45 µm filter. When growing to 40 ~ 60% confluence, SW1116 and SW480 cells were infected with viral supernatants. Forty‐eight hours later, 2 μg/ml puromycin was added to the medium and the stable cells were selected.

### 2.4 Coimmunoprecipitation, western blot and antibodies

The transient transfection was performed using Lipofectamine 2000 (Invitrogen, *Cat#* 11668019) as described.^16^ Plasmids for Myc‐Ajuba, Flag‐Twist and/or their truncation mutants were transiently transfected into 293T cells. After 24 hours, cells were collected and lysed in buffer containing 20 mmol/L Tris (pH 7.5), 150 mmol/L NaCl, 2.5 mmol/L EDTA, DTT and protease inhibitor mixture. The whole‐cell lysates were precleared with normal mouse IgG beads (Sigma, *Cat#*A0919) and Co‐IP assays were performed with either α‐Myc or α‐Flag antibodies. In all Co‐IP experiments, 3% inputs were loaded as controls for Western blot. Western blot assay was performed according to the previous description.^8^ The antibodies used for Co‐IP, Western blot and chromatin immunoprecipitation are as follows: mouse monoclonal anti‐Myc (Invivogen, *Cat#*MA1‐980, 1:2000), anti‐Flag (Sigma, *Cat#*SAB4301135, 1:2000), anti‐HA (Cell Signaling, *Cat#*3724, 1:1000), anti‐CBP (Cell Signaling, *Cat#*7389, 1:1000), anti‐acetyl‐H3(Millipore, *Cat#* 06‐559), anti‐Ajuba (Cell Signaling, *Cat#* 4897, 1:1000) and anti‐Twist (Proteintech, *Cat#*25465, 1:1000).

### 2.5 Quantitative real‐time PCR (qRT‐PCR) and reverse transcription PCR (RT‐PCR)

Total RNA from cells was prepared using TRIzol reagent (Life Sciences) following the manufacturer's protocol. Complementary DNA was synthesized with 2 µg of total RNA using SuperScript Ⅳ RT Reagent Kit (Invitrogen, *Cat#*18091050). qRT‐PCR assays were carried out using the SYBR Green PCR Master Mix (Applied Biosystems, Foster City, CA). Semi‐quantitative PCR assays were used to detect the level of N‐cadherin and β‐actin. The level of β‐actin was used as the endogenous control. The specific primers used were as follows: 5'‐TACCAGGACGAGCTAACAGC‐3' (forward) and 5'‐GCACTTGATA‐CAGGTGCCGAA‐3' (reverse) for *Ajuba*; 5'‐GCCGGAGACCTAGATGT‐CATT −3' (forward) and 5'‐TTTTAAAAGTGCGCCCCACG‐3' (reverse) for *Twist*; 5'‐ACAGTGGCCACCTACAAAGG‐3' (forward) and 5'‐CCGAG‐ATGGGGTTGAT‐AATG‐3' (reverse) for *N‐cadherin*; and 5'‐TCACCAACTG‐GGACGACAT‐3' (forward) and 5'‐CACAGCCTGGATAGCAACG‐3' (reverse) for *ꞵ‐actin*. The fold changes were shown as means ± SD in three independent experiments.

### 2.6 Chromatin immunoprecipitation

SW480 cells were stably transfected with the indicated plasmids and subjected to ChIP assays. The method has been described previously.^16^ The chromatin was sonicated to fragments ranging from 500 to 800 bp in size. Immunoprecipitation was performed using the specific anti‐human antibodies against Ajuba, Twist, CBP and acetyl‐H3. An equal amount of normal rabbit IgG (Cell Signaling, *Cat#*7389) was used as a negative control. The precipitated DNA fragments were detected by qRT‐PCR with primer set 1: 5'‐GGAAGCAGAGCAGTTTACGC‐3' and 5'‐ATCGGCTGCTTAGTCTGG‐AA‐3'. The 220‐bp amplicon flanks the E‐box located in intron 1 of *N‐cadherin* gene. Primer set 2: 5'‐TGCGTCCTTAGTTTGCTGTG‐3' and 5'‐GGGCAGGAACTTGATTGGTA‐3', was used to amplify a 187‐bp fragment flanking 5000‐bp region upstream of the transcription start site.

### 2.7 Dual‐luciferase reporter assay

293T cells were seeded with a 12‐well plate. The pRL‐SV40 vector (20 ng) and pGL3‐N‐cadherin‐Luc reporter (200 ng), along with Ajuba‐ and/or Twist‐ and CBP‐encoding plasmids, were transiently cotransfected into 293T cells with Lipofectamine 2000 reagent (Invitrogen, *Cat#*11668019). Twenty‐four hours post‐transfection, cells were harvested and the luciferase activity assays were carried out following the kit's instructions (Promega, *Cat#*E1910). Three independent experiments were carried out in triplicate.

### 2.8 Statistical analysis

Experiments were performed at least three times, and results were shown as mean ± SD, Statistical analysis was performed using Student's two‐tailed exact *t* test, and *P* values less than 0.05 were considered statistically significant.

## 3 RESULTS

### 3.1 The LIM region of Ajuba binds to Twist

**FIGURE 1:**
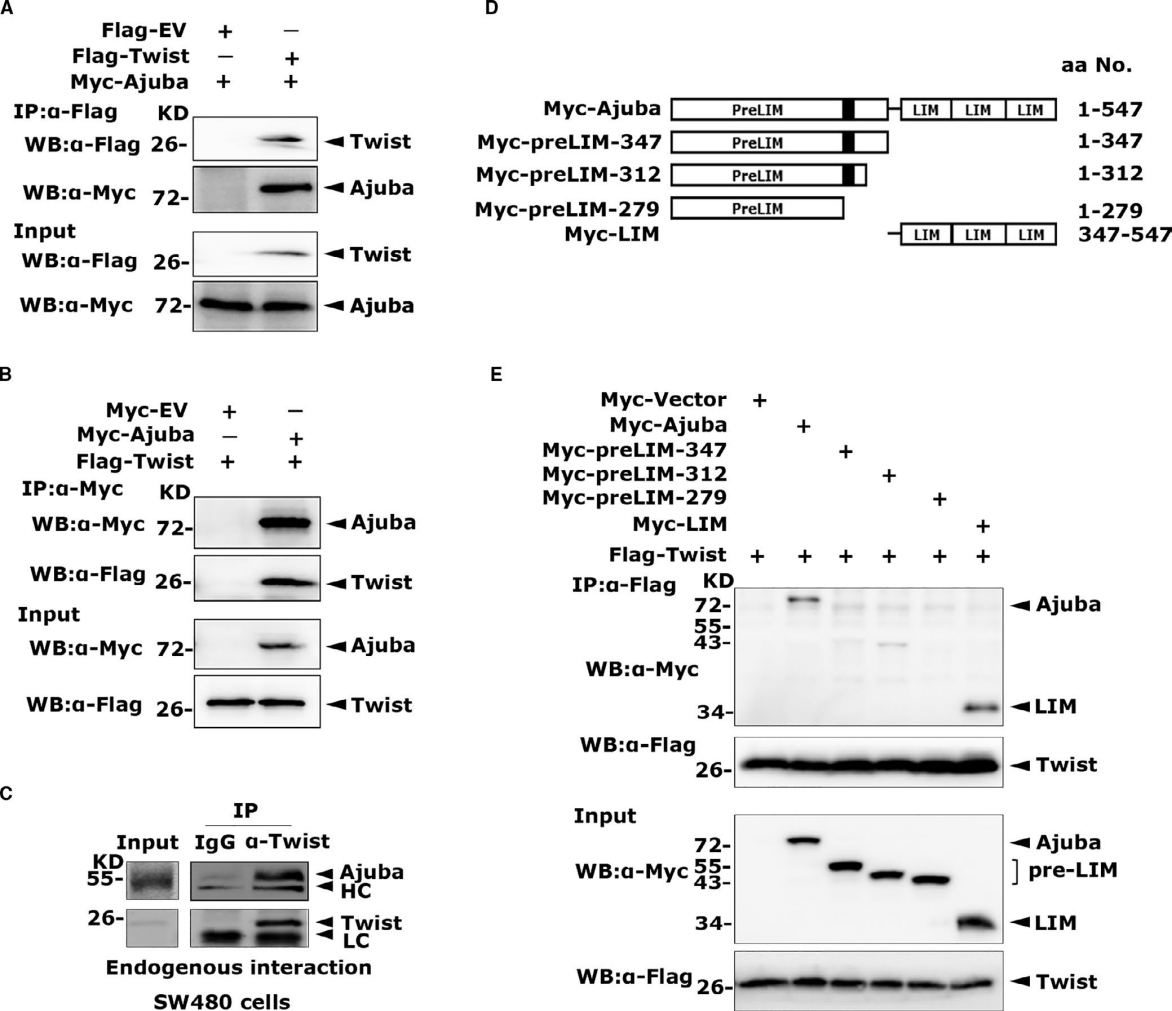
The LIM region of Ajuba binds Twist

To further examine the mechanism of Ajuba promoting tumour metastasis, Twist was chosen for the assay of Ajuba‐interacting proteins. We transfected Flag vector or Flag‐Twist and Myc‐Ajuba into 293T cells, and Co‐IP assay with anti‐flag M2 beads showed that Twist can pull down Ajuba (Figure 1A). Conversely, Ajuba can immunoprecipitate Twist with an anti‐Myc antibody (Figure 1B). The reciprocal coimmunoprecipitation experiments indicated that Ajuba and Twist can robustly interact with each other. To verify the endogenous interaction between Ajuba and Twist, the whole‐cell lysates from SW480 cells were incubated with Twist antibody, and the co‐eluted Ajuba protein was detected. The results revealed that endogenous Twist pulled down Ajuba (Figure 1C). These finding suggested that Twist can interact with Ajuba both in vitro and in vivo .

To map which region in Ajuba participates in interaction with Twist, we constructed the plasmids encoding Myc‐tagged Ajuba and four truncated mutants (Figure 1D). PreLIM‐279, PreLIM‐312, PreLIM‐347 and LIM were coexpressed together with plasmids encoding Flag‐Twist in 293T cells, and the Co‐IP assays were performed with anti‐Flag M2 beads. Obviously, the truncations preLIM‐279 and preLIM‐347 did not pull down Twist, and preLIM‐312 displayed weak binding ability to Twist, whereas the LIM domain alone displayed the same binding capability to Twist as the full‐length Ajuba (Figure 1E). Taken together, these results demonstrated that the LIM region of Ajuba binds to Twist.

### 3.2 The Twist box region of Twist binds to Ajuba

**FIGURE 2:**
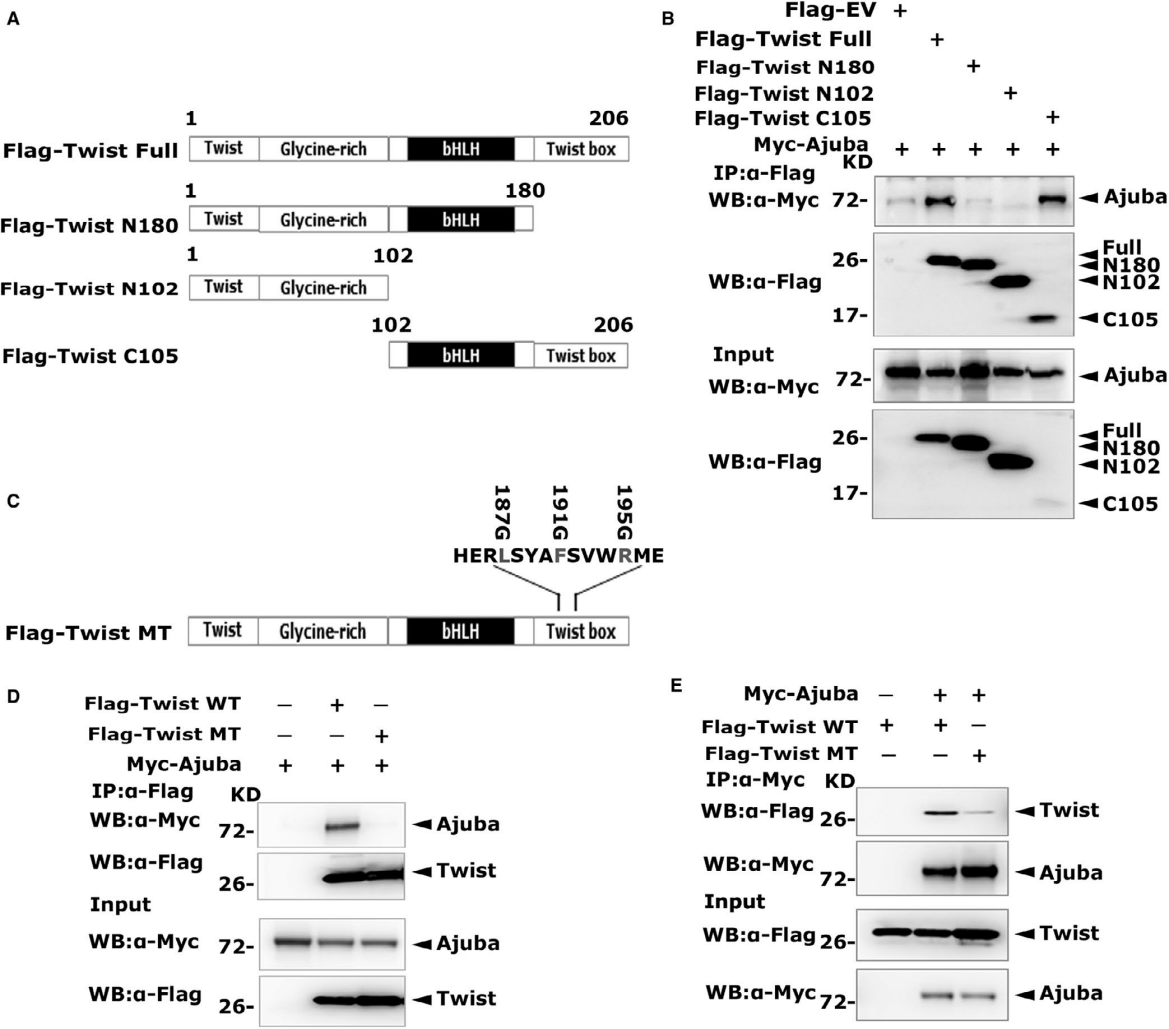
The Twist box region of Twist binds Ajuba

Structurally, Twist protein comprises four different domains: (1) a highly conservative Twist domain; (2) a glycine‐abundant domain; (3) a basic helix‐loop‐helix motif; and (4) the Twist box region (or WR region).^17^ To delineate the motifs in Twist engaged in the interaction, we recombined truncations of Twist (Figure 2A). The Co‐IP experiments were carried out with anti‐Flag antibody. As shown in Figure 2B, the Twist mutants N180 and N102 did not pull down Ajuba, whereas the Twist C105 displayed the similar binding capability to Ajuba as the full‐length Twist did. To further consolidate that the Twist box motif is pivotal for the interplay, we mutated the three conservative bases of Twist box^18^ (Figure 2C). Next, we cotransfected Myc‐Ajuba and Flag‐Twist wild type (WT) or mutant (MT) into 293T cells, and Co‐IP experiment was performed with Flag antibody. Interestingly, the wild‐type Twist, not the mutant one, pulled down Ajuba. The other way round, we carried out the Co‐IP assays with Myc antibody. The results showed that Ajuba intensely bound the wild Twist, but weakly bound the mutated one (Figure 2D&E). These findings suggested that the Twist box region was essential for the interaction between Ajuba and Twist.

### 3.3 Ajuba and Twist synergistically bind to Twist target promoter region to transactivate *N‐cadherin* expression

**FIGURE 3:**
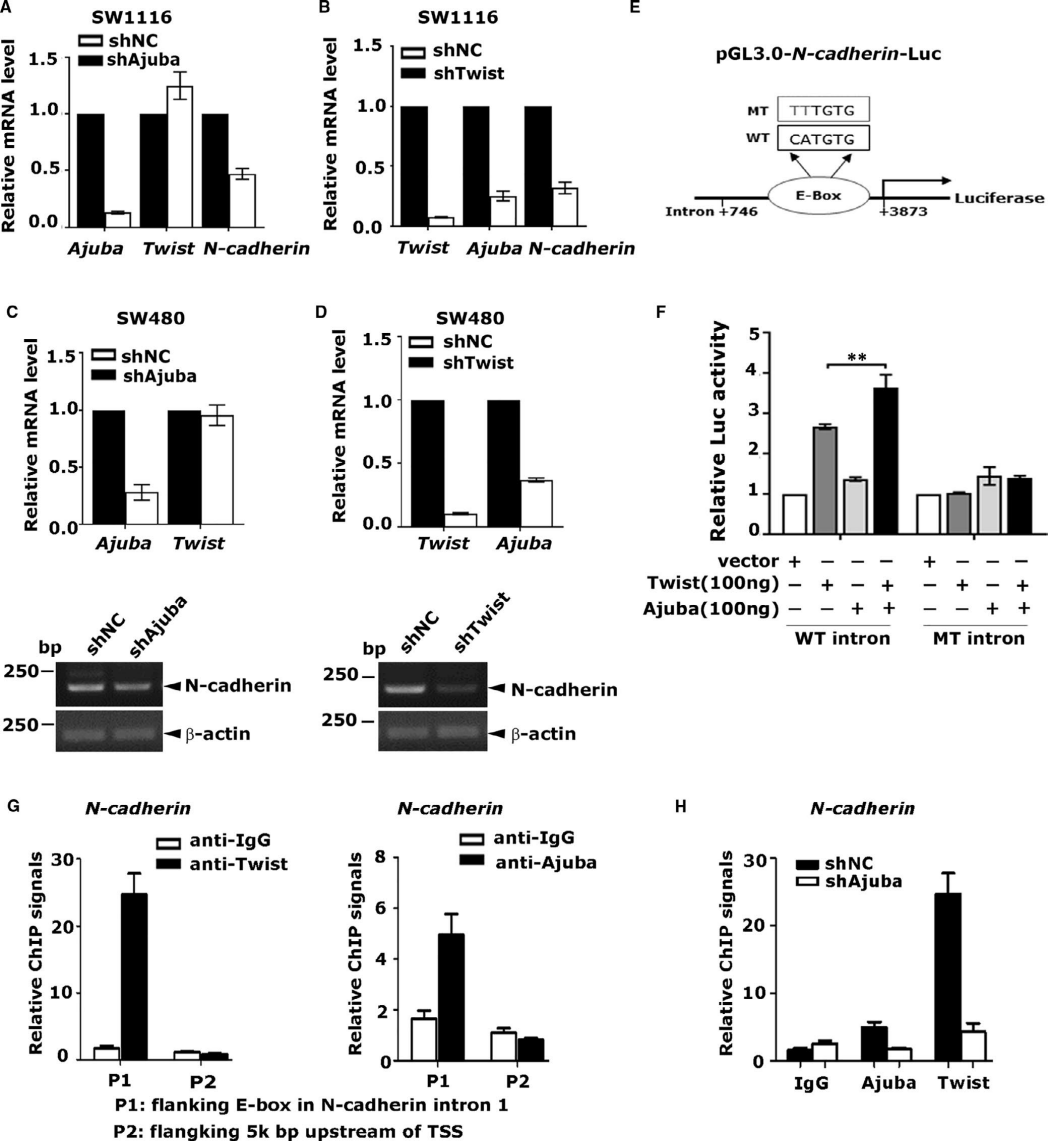
Ajuba and Twist cooperatively bind to Twist target promoter

The finding described above prompted us to wonder whether Ajuba affects Twist‐mediated transcriptional activity. Next, SW1116 and SW480 cells were stably depleted using lentiviral shRNA (Figure 3A). The total RNAs were extracted and reverse‐transcribed for qRT‐PCR and RT‐PCR analyses. During cancer metastasis, Twist is considered as a crucial transcription factor in regulating the gene expression of *N‐cadherin*.^13^ Here, *N‐cadherin* was selected to assess the transcription activity of Twist. In the absence of Ajuba, the expression of *N‐cadherin* in SW1116 and SW480 was decreased in different degrees (Figure 3A‐C). Notably, depletion of Ajuba in SW1116 and SW480 cells did not apparently affect the expression of Twist (Figure 3A‐C), indicating that deregulated *N‐cadherin* expression is not caused by the fluctuation of Twist expression. Surprisingly, the knockdown of Twist in SW1116 and SW480 cells decreased the expression of Ajuba, suggesting that Twist might affect the transcription of Ajuba (Figure 3B‐D). Collectively, these results manifested that Ajuba is a vital coactivator for Twist‐related transcriptional activity.

To investigate whether Ajuba directly regulates Twist downstream targets, a human N‐cadherin promoter containing a functional Twist E‐box (+2627‐ +2632) was linked to pGL3‐basic luciferase plasmid to produce an N‐cadherin‐Luc reporter^14^ (Figure 3E). Next, N‐cadherin‐Luc reporter (N‐cad‐Luc), along with plasmids Flag‐Twist and Myc‐Ajuba, was cotransfected into 293T cells and the luciferase activity was determined and normalized to Renilla luciferase activity. Ajuba or Twist alone weakly or modestly increased N‐cad‐Luc activity, whereas coexpression of Ajuba and Twist robustly increased N‐cad‐Luc activity. Additionally, mutagenesis of the E‐box sites at +2627 region resulted in not only the loss of transactivation ability of Twist to induce N‐cadherin promoter activity but also that of the synergy (Figure 3F). Taken together, these data suggested that Ajuba worked in coordination with Twist to transactivate N‐cadherin expression.

Next, to verify whether Twist and Ajuba directly bind the endogenous E‐box region of N‐cadherin, the ChIP assay was performed with Twist and Ajuba antibodies in SW480 cells. The precipitated DNA was amplified by qRT‐PCR using primers flanking the E‐box region of N‐cadherin, and the region ~5000bp upstream of N‐cadherin transcription start site was used as a negative control. Our results showed that both Ajuba and Twist bound to the E‐box region of N‐cadherin, suggesting that Ajuba formed a complex with Twist to bind to the E‐box region of N‐cadherin. In addition, knockdown of Ajuba greatly reduced the enrichment of N‐cadherin promoter DNA by Twist antibody (Figure 3H). Collectively, these results indicate that the presence of Ajuba is essential for the binding of Twist to the target promoter region, and Ajuba augments the binding ability of Twist to N‐cadherin promoter.

### 3.4 Ajuba bridges Twist and CBP to regulate target gene transcription

**FIGURE 4:**
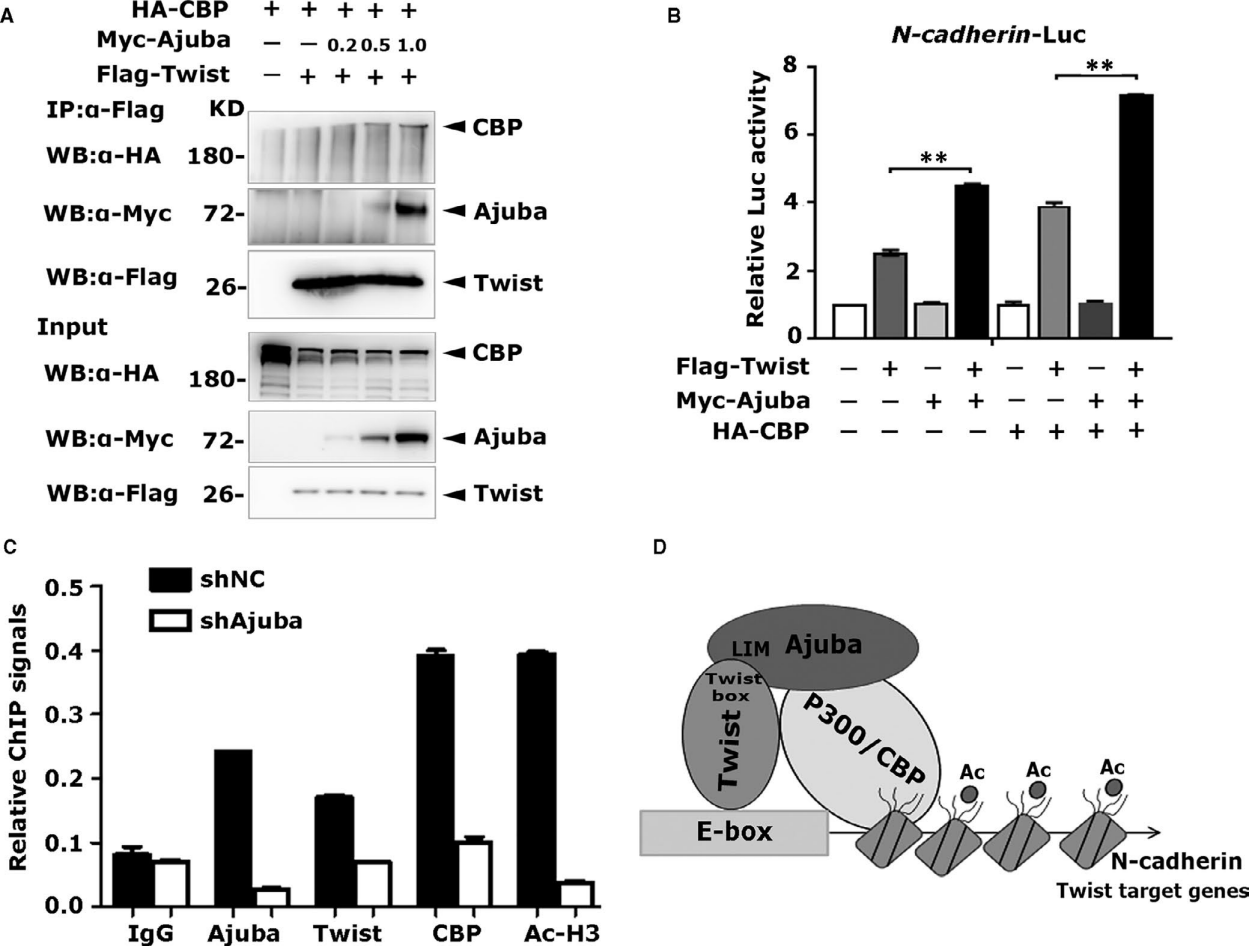
Ajuba recruits CBP to Twist and its target chromatins

It was reported that Ajuba can enhance the interaction between p300/CBP and PPARγ to activate the latter target gene transcription.^9^ Here, we questioned whether Twist, Ajuba and p300/CBP could form functional ternary complexes. To answer this question, the plasmids Flag‐Twist and HA‐CBP, together with an increasing amount of Myc‐Ajuba, were cotransfected into 293T cells, and Co‐IP experiment was performed with Flag antibody. As shown in Figure 4A, Twist did not pull down CBP, while Ajuba readily immunoprecipitated CBP. Moreover, increased Ajuba made Twist precipitate more Ajuba together with CBP, indicating that Ajuba might function as an adaptor protein to assemble the complex of Twist, Ajuba and CBP. To test the function of Twist, Ajuba and CBP complex in transactivating Twist target promoter, luciferase reporter analysis on N‐cadherin promoter was performed in 293T cells. Our results showed that Twist stimulated weak promoter activity; a combination of Twist and Ajuba, or Twist and CBP modestly induced the N‐cadherin activity, whereas combination of Twist, Ajuba and CBP resulted in the strongest N‐cadherin activity (Figure 4B), indicating that a synergistic effect in Twist, Ajuba and p300/CBP exists at the target promoter region.

To further verify the assembling of Ajuba, Twist and CBP ternary complex at endogenous chromatins, the ChIP assay was carried out in SW480‐shNC and SW480‐shAjuba cells using Twist, Ajuba, CBP and H3‐Ac antibodies respectively. We used the primer sets flanking the E‐box site to measure precipitated DNA fragments by qPCR. In SW480‐shNC cells, Twist, Ajuba and CBP were identified to bind to Twist target promoter in different degrees, and acetylated histone maintained at the middle level represented by H3‐Ac antibody (Figure 4C). Surprisingly, loss of Ajuba triggered strikingly decreased binding of Twist and CBP at the target chromatin region accompanying remarkably decreased histone acetylation at the same locus (Figure 4D). Taken together, all data show that Ajuba is indispensable for Twist to efficiently recruit p300/CBP to its target chromatin region.

## 4 DISCUSSION

Herein, we found that Ajuba can interact with the Twist box of Twist via its LIM domain. Furthermore, we demonstrated that Ajuba recruits CBP/p300 and Twist to the target chromatins to augment histone acetylation to induce the transcription of N‐cadherin. Together, our results showed that Ajuba is a key coactivator for Twist.

Structurally, Ajuba contains the N‐terminal preLIM domain and the C‐terminal three tandem LIM domains. The LIM domains supply a scaffold for various protein‐protein interplays.^5^, ^19^, ^20^ The NLS in LIM region and the NES in preLIM enable Ajuba to shuttle between cytoplasm and nucleus. As Twist is a transcription factor exerting its function in the nucleus and our data showed Ajuba interacts with Twist, we assumed that Ajuba could translocate into the nucleus to bind Twist for synergistic regulation.

Twist, a major marker of EMT, can facilitate cell motility and invasive activity, and enhances some features of cancer cells via regulating a series of target gene expression.^12^ Deletion of Twist attenuated cancer cell migration and invasion abilities.^21^ The Twist box domain is highly conservative among different species and identified to be obligatory for the transactivation of downstream targets. Additionally, three residues Leu‐187, Phe‐191 and Arg‐195 in the Twist box are necessary for transactivation.^18^ Evidence showed that the ectopic expression of Twist mutant (Twist‐F191G) triggers defection of EMT in Myc‐Cap cells.^17^ Our results demonstrated that the mutation of the three conserved residues in the Twist box strikingly inhibits the binding to Ajuba, suggesting that Ajuba might be required for transactivation. Collectively, these results supplied new mechanism for Ajuba‐ and Twist‐mediated metastasis regulation.

Mechanistically, the previous investigation showed that Ajuba can function as a coactivator for PPARγ and ERα by recruiting CBP/P300.^9^, ^22^ In this paper, we further verified that Ajuba acted as a scaffold protein to recruit Twist and CBP to target chromatin and collaboratively activate Twist target gene expression.

In summary, our results not only enriched the research on the tumour metastasis mechanism of Twist and Ajuba, but suggested that Ajuba/Twist/CBP/p300 pathway may be a new target for therapeutics to cancer metastasis.

## CONFLICT OF INTEREST

The authors declare no conflict of interest.

## AUTHOR CONTRIBUTION

**Zhaoxia Wu :** Conceptualization (equal); Methodology (equal). **Xiuqun Zou** **:** Methodology (equal); Supervision (equal). **Ying Xu:** Resources (equal); Validation (equal). **Fengli Zhou:** Investigation (equal). **Rong Kuai:** Investigation (equal). **Ji Li:** Software (equal). **Daming Yang:** Data curation (equal); Formal analysis (equal). **Yimin Chu:** Supervision (equal); Writing‐original draft (equal). **Haixia Peng:** Data curation (equal); Funding acquisition (equal); Writing‐review & editing (equal).

## Data Availability

The data that support the findings of this study are available from the corresponding author upon reasonable request.
